# A checklist of chiggers from Brazil, including new records (Acari: Trombidiformes: Trombiculidae and Leeuwenhoekiidae)

**DOI:** 10.3897/zookeys.743.22675

**Published:** 2018-03-14

**Authors:** Fernando de Castro Jacinavicius, Ricardo Bassini-Silva, Jairo Alfonso Mendoza-Roldan, Almir Rogério Pepato, Ronald Ochoa, Cal Welbourn, Darci Moraes Barros-Battesti

**Affiliations:** 1 Laboratório Especial de Coleções Zoológicas, Instituto Butantan, São Paulo, SP; Brazil; 2 Departamento de Medicina Veterinária Preventiva e Saúde Animal, FMVZ-USP, São Paulo, SP, Brazil; 3 Departamento de Zoologia, Instituto de Ciências Biológicas, Universidade Federal de Minas Gerais, Belo Horizonte, MG, Brazil; 4 Systematic Entomology Laboratory, United States Department of Agriculture, Agricultural Research Service, Beltsville, MD, USA; 5 Florida State Collection of Arthropods, Division of Plant Industry, Florida Department of Agriculture and Consumer Services, Gainesville, FL, USA; 6 Departamento de Patologia Veterinária, Faculdade de Ciências Agrárias e Veterinárias-UNESP, Jaboticabal, SP, Brazil

**Keywords:** Brazil, Chigger mites, Cricetidae, Didelphidae, distribution

## Abstract

A checklist of the family Trombiculidae and Leeuwenhoekiidae is presented, containing 63 species in 30 genera of chiggers from 80 different hosts and 146 localities in Brazil. The type locality and depository are provided, including new locality and host records for the country.

## Introduction

The mites known as “chiggers” or “chigger mites” belong to three families: Trombiculidae Ewing, 1944, Leeuwenhoekiidae Womersley, 1944, and Walchiidae Ewing, 1946 *sensu* Wen (1999). The larvae are ectoparasites of vertebrates, and during the parasitism, they liquefy and suck the host’s epithelial tissue by the enzymatic action of their saliva. Blood is not ingested in this process, but some blood cells can be found in the gut of the mite (Jones 1950). Deutonymphs and adults are free living predators (Crossley 1960).

Studies of the biology and taxonomy of these mites were intensified during World War II, thus increasing considerably the number of known species. Soldiers were attacked in encampments and contracted rickettsioses from infected larvae (Wharton and Fuller 1952). According to these authors, in the front of the situation, the public health departments were faced with the need for cataloging studies, description, morphology, and biology of these mites. Although they are considered potential vectors of pathogens, the role of these mites in the epidemiology of rickettsiosis is unknown in the Neotropical region (Azad and Beard 1998, Poinar and Poinar 1998).

In South America, the countries that have a checklist of chiggers species are: Venezuela (Brennan and Reed 1975), Peru (Brennan and Jones 1961), Surinam (Brennan and Lukoschus 1971) and recently Chile (Stekolnikov and González-Acuña 2015). Ewing (1931) compiled the first list of chigger mites from the New World, eight species from Brazil. Wharton and Fuller (1952) increased the number by eight genera and 18 species. Fonseca (1955) reported the first human hosts for Brazil. Brennan and Goff (1977) reported 87 chigger genera for the Nearctic and Neotropical regions, of which 18 were recorded from Brazil.

The Brazilian territory is composed by five regions (north, northeast, central-west, southeast and south), 26 states and one federal district (Contel 2014). Between the 1970s and 1990s, new species were described by various researchers from specimens collected primarily in the northern and central-western parts of the country, but have never been summarized (Stekolnikov and González-Acuña 2015). Therefore, here we are compiling a checklist of species from Brazil, including new hosts and localities.

## Materials and methods

In the checklist, the taxa comprise two families, Trombiculidae and Leeuwenhoekiidae. The genera and species are arranged alphabetically, in the follow format: Genus - Valid name, author and data; Species – Checklist number, valid name of species, author, date, type locality, host, and type depository, in parenthesis are the typification and the collection number, when available. In cases where the original host name has changed, the current host name is listed with the original host name in parenthesis. The host species were updated based on the IUCN (2017).

The following abbreviations were used:

**BPBM** USA, Hawaii, Honolulu, Bernice Pauahi Bishop Museum;

**FMNH** USA, Illinois, Chicago, Field Museum of Natural History Chicago (cited in original description as CNHM);

**IBSP** Brazil, São Paulo, São Paulo, Instituto Butantan;

**MNRJ** Brazil, Rio de Janeiro, São Cristovão, Universidade do Rio Janeiro, Museu Nacional;

**MZSP** Brazil, São Paulo, São Paulo, Universidade de São Paulo;

**NHMUK** United Kingdom, London, The Natural History Museum [formerly British Museum (Natural History)] (cited in original description as BMNH or BM);

**OMNH** USA, Oklahoma, Norman, University of Oklahoma, Oklahoma Museum of Natural History;

**OSAL** USA, Ohio, Columbus, Ohio State University Acarology Laboratory;

**RMNH** Netherlands, Leiden, Naturalis Biodiversity Centre [formerly Rijksmuseum van Natuurlijke Historie];

**SAM** Australia, South Australia, Adelaide, South Australian Museum;

**USNM** USA, Washington D.C., National Museum of Natural History, [formerly United States National Museum] which is housed at the Systematic Entomology Laboratory (BARC-USDA-ARS), cited in original descriptions as NMNH.

Details of the records in Brazil were provided based on the bibliographic review, followed to the new records for the country, when available. The new records from the checklist are based on the material deposited in the IBSP collection. Some of these species were also reported outside Brazilian territory. These recordings of literature were included in other records.

In addition, a list of chiggers from Brazil is summarized with their respective hosts (Appendix 1) and a list of vertebrate hosts and their respective chiggers and the published information from Brazil (Appendix 2).

## Results

A Brazilian checklist with eight species of the family Leeuwenhoekiidae and 55 of the family Trombiculidae, totaling 63 chigger species in 30 genera from approximately 70 different hosts is presented. Some regions just have one or two chigger records and most of these records are represented by the type series. Species of the family Walchiidade were never recorded in the Brazilian territory until this work.

The chigger species *P. aemulata*, *Q. flochi*, *Q. mirandae*, and *Q. trapezoides* are reported for the first time in Brazil, and new records of locality for *T. bakeri* and new localities and hosts for the species *C. spinosus*, *E. tinami*, *K. brasiliensis*, *Q. pazca*, and *S. tamarina* are provided too.

## Checklist of larvae chigger species (in alphabetical order) recorded from Brazil

### Family LEEUWENHOEKIIDAE Womersley, 1944

**Genus *Apolonia* Torres & Braga, 1938: 172**

**1. *Apolonia tigipioensis* Torres & Braga, 1938: 172**; Tigipió, Pernambuco state, Brazil, ex *Gallus gallus domesticus*; IBSP (syntype, n° 1954).

**Records from Brazil.** In addition to the type data, Carneiro (1949, 1952) reported *A. tigipioensis* parasitizing humans and *Nothura maculosa cearensis* (Tinamiformes) in Limoeiro (Pernambuco). This chigger was also found parasitizing the birds *Struthio camelus* (Struthioniformes) and *Passer domesticus* (Passeriformes) in Petrolina (Pernambuco) (Ornelas-Almeida et al. 2007).

**Other records.** Venezuela (Brennan and Reed 1975).

**Genus *Hannemania* Oudemans, 1911: 137**

**2. *Hannemania hepatica* Fonseca, 1936: 30**; Butantan, São Paulo, São Paulo state, Brazil; ex. *Leptodactylus latrans* (= *L. ocellatus*); IBSP (holotype, n° 31).

**Records from Brazil.** Only the type data.

**3. *Hannemania hylodeus* (Oudemans, 1910): 88**; Brazil, ex *Hylodes* sp.; RMNH (n° 6247).

**Records from Brazil.** Only the type data, no locality information was provided by the author.

**4. *Hannemania newsteadi* Sambon, 1928: 127**; Urucum, Mato Grosso do Sul state, Brazil, ex *Hyla rubra*; NHMUK.

**Records from Brazil.** Only the type data.

**5. *Hannemania stephensi* Sambon, 1928: 127**; Tombador, Mato Grosso state, Brazil, ex *Pristimantis conspicillatus* (= *Hylodes conspicillatus*); NHMUK.

**Records from Brazil.** Only the type data.

**Genus *Leeuwenhoekia* Oudemans, 1911: 137**

**6. *Leeuwenhoekia verduni* (Oudemans, 1910): 88**; southern Brazil; ex. *Didelphis* sp., “opossum” RMNH (n° 6250).

**Records from Brazil.** Only the type data, this species was described from an unknown location in southern Brazil.

**Genus *Whartonia* Ewing, 1944: 102**

**7. *Whartonia nudosetosa* (Wharton, 1938): 142**; Cave near Oxkutzcab, Yucatan, Mexico, ex *Artibeus jamaicensis yucatanicus* and “*Peteropteryx canina canina*”; USNM (n° 1264).

**Note.** The correct host name is *Peropteryx macrotis*, originally described as *Verpertillio caninus*.

**Records from Brazil.** Silveira et al. (2015) reported this species from Medina and São José da Safira (Minas Gerais state) on *Carollia perspicillata* (Chiroptera).

**Other records.** Costa Rica (Webb and Loomis 1977); Guatemala (Brennan and Dalmat 1960); Jamaica (Brennan 1953); Nicaragua (Webb and Loomis 1977); Mexico (Wharton 1938, Hoffmann 1949, Loomis 1969, Webb and Loomis 1977); Surinam (Brennan and van Bronswijk 1975); Trinidad (Brennan and Jones 1960, Brennan 1967) and Venezuela (Reed and Brennan 1975).

**8. *Whartonia pachywhartoni* Vercammen-Grandjean, 1966: 282**; Lagoa Santa, Minas Gerais state, Brazil, ex *Micronycteris megalotis*, USNM (holotype, n° 10462/6).

**Records from Brazil.** In addition to the type data, *W. pachywhartoni* was found in São José da Safira (Minas Gerais) parasitizing the bat *C. perspicillata* (Silveira et al. 2015).

### Family TROMBICULIDAE Ewing, 1944

**Genus *Aitkenius* Brennan, 1970: 1694**

**9. *Aitkenius vellosus* Brennan, 1970: 1695**; Belém, Pará state, Brazil, ex *Proechimys guyannensis*, USNM (holotype and paratypes, n° 48876), FMNH (paratypes) and NHMUK (paratypes).

**Records from Brazil.** In addition to the type data, *A. vellosus* was also found at the same locality, on *Hylaeamys megacephalus* (Rodentia), and in Bragança (Pará) on the water rat *Nectomys squamipes* (Rodentia) (Brennan 1970d).

**Genus *Arisocerus* Brennan, 1970: 32**

**10. *Arisocerus amapensis* Brennan, 1970: 32**; Serra do Navio, Amapá state, Brazil, ex *Euryoryzomys macconnelli*; USNM (holotype and paratypes, n° 49368).

**Records from Brazil.** In addition to the type data, this species was also found at the same locality, on *H. megacephalus*, and in Belém (Pará) parasitizing *H. megacephalus* and *P. guyannesis* (Brennan 1970c).

**Other records.** Suriname (Brennan 1970c, Brennan and van Bronswijk 1975) and Venezuela (Brennan and Reed 1975).

**11. *Arisocerus hertigi* (Brennan & Jones, 1964): 308**; Sommerfield, Paraguay, ex “agouti”; USNM (holotype and paratypes, n° 43427), FMNH (paratypes), OSAL (paratypes), BPBM (paratypes).

**Records from Brazil.** This species was found in Brasília (Federal District), parasitizing the marsupial *Didelphis albiventris* (Didelphimorphia) (Goff and Gettinger 1989). In addition, *A. hertigi* was found on the Archipelago Moleques do Sul (Santa Catarina) parasitizing *Cavia intermedia* (Rodentia) (Regolin et al. 2015).

**Other records.** Paraguay (Brennan and Jones 1964a).

**Genus *Blankaartia* Oudemans, 1911: 123**

**12. *Blankaartia shatrovi*Bassini-Silva & Barros-Battesti, 2016: 83**; São Paulo, São Paulo state, Brazil, ex “bird”; IBSP (holotype and paratypes, n° 367).

**Records from Brazil.** In addition to the type data, this species was also found in Coronel Pacheco (Minas Gerais) parasitizing *Trichothraupis melanops* (Passeriformes) (Bassini-Silva et al. 2016).

**13. *Blankaartia sinnamaryi* Floch & Fauran, 1956: 3**; Sinnamary, French Guiana, ex *Homo sapiens*.

**Records from Brazil.** This species was found in Paraty (Rio de Janeiro) parasitizing *Manacus manacus* (Passeriformes), in Juiz de Fora (Minas Gerais) parasitizing *Picumnus cirratus* (Piciformes), *Tachyphonus coronatus*, *Thamnophilus caerulescens* and *Turdus rufiventris* (Passeriformes), and in Coronel Pacheco (Minas Gerais) parasitizing *Conopophaga lineata* and *Turdus albicollis* (Passeriformes) (Bassini-Silva et al. 2016).

**Other records.** Costa Rica (Arnold 1970, Stekolnikov et al. 2007), Cuba (Daniel and Stekolnikov 2003), French Guiana (Floch and Fauran 1956), Jamaica (Brennan 1953), Panama (Brennan and Yunker 1966), Peru (Brennan and Jones 1961a), Surinam (Brennan and van Bronswijk 1975), Trinidad (Brennan and Jones 1960), USA (Brennan 1965, Spalding et al. 1997) and Venezuela (Brennan and Reed 1975).

**Genus *Buclypeus* Brennan, 1972: 1178**

**14. *Buclypeus catatonus* Brennan, 1972: 1178**; Belém, Pará state, Brazil, ex *Proechimys guyannensis*; USNM (holotype and paratypes, n° 49322), NHMUK (paratypes), BPBM (paratypes) and FMNH (paratypes).

**Records from Brazil.** Only the type data.

**15. *Buclypeus ignotus* (Brennan, 1971): 214**; Belém, Pará state, Brazil, ex *Proechimys guyannensis*, USNM (holotype and paratypes, n° 49322), NHMUK (paratypes) and FMNH (paratypes).

**Records from Brazil.** Only the type data.

**Genus *Caamembecaia* Gazêta, Amorim, Bossi, Linhares & Serra-Freire, 2006: 137**

**16. *Caamembecaia gratiosus* Gazêta, Amorim, Bossi, Linhares & Serra-Freire, 2006: 137**; Itatiaia National Park, Rio de Janeiro state, Brazil, ex *Trinomys gratiosus*; MNRJ (holotype, n° 67498).

**Records from Brazil.** Only the type data.

**Genus *Chiroptella* Vercammen-Grandjean, 1960: 469**

**17. *Chiroptella*** (***Oudemansidium***) ***australis* (Brennan, 1970): 810**; Pedras Negras, Rondônia state, Brazil, ex *Nyctinomops laticaudatus* (= *Tadarida laticaudata)*; USNM (holotype and paratypes, n° 50192), NHMUK (paratypes) and FMNH (paratypes).

**Records from Brazil.** Only the type data.

**Other records.** Venezuela (Brennan and Reed 1975).

**Genus *Colicus* Brennan, 1970: 271**

**18. *Colicus brasiliensis* Goff, Whitaker & Dietz, 1983: 185**; São Roque de Minas, Minas Gerais state, Brazil, ex *Oligoryzomys formesi* (= *Oryzomys fornesi*) and *Cerradomys subflavus* (= *Oryzomys subflavus*); BPBM (holotype, n°12711 and paratypes).

**Records from Brazil.** Only the type data.

**19. *Colicus icomi* Brennan, 1970: 271**; Serra do Navio, Amapá state Brazil, ex *Proechimys guyannensis*; USNM (holotype and paratypes, n° 49342).

**Records from Brazil.** Only the type data.

**Other records.** Surinam (Brennan and van Bronswijk 1975).

**20. *Colicus spinosus* Goff & Gettinger, 1989: 554**; Brasília, Federal District, Brazil, ex *Gracilinannus agilis*; MZSP (holotype), BPBM (paratypes), OMNH (paratypes) and USNM (paratypes).

**Records from Brazil.** Only the type data.

**New record.** IBSP 11116 (1 larva), Condomínio Vila Verde, Itapevi, São Paulo, state of São Paulo, 29-XI-2012, *Monodelphis* sp. (Didelphimorphia) (#VV 105).

**Genus *Euschoengastia*Ewing 1938: 293**

**21. *Euschoengastia trouessarti* (Oudemans, 1910): 87**; southern Brazil; ex *Didelphis* sp.; RMNH.

**Records from Brazil.** Only the type data, this species was described from an unknown location in southern Brazil.

**Genus *Eutrombicula* Ewing, 1938: 293**

**22. *Eutrombicula alfreddugesi* Oudemans, 1910: 84**; México, ex *Homo sapiens*; RMNH.

**Records from Brazil.** This species was found in Correntes (Mato Grosso) parasitizing the snake, *Xenodon merremii* and a human in São Paulo (São Paulo) (Fonseca 1932a). This species was also found in Unaí (Minas Gerais) and Pirenópolis (Goiás) parasitizing *Tropidurus oreadicus*, *Tropidurus itambere* and *Tropidurus torquatus* (Squamata) (Carvalho et al. 2006). There are additional records from Chapada do Araripe (Ceará) on *Tropidurus hispidus* (Squamata) (Delfino et al. 2011) and from Morro do Chapéu (Bahia), on *Tropidurus hispidus*, *Tropidurus cocorobensis*, *Tropidurus semitaeniatus* and *Tropidurus erythrocephalus* (Menezes et al. 2011). Recently this species was found parasitizing goats in Maranhão state (Faccini et al. 2017).

**Other records.** Bolivia (Brennan 1970a); Costa Rica (Arnold 1970); Cuba (Daniel and Stekolnikov 2004); USA (Rohani and Cromroy 1979); Guatemala (Brennan and Dalmat 1960); Mexico (Oudemans 1910, Wharton 1938, Pearse et al. 1936, Hoffmann 1949, Jenkins 1949, Kroman et al. 1967, Loomis 1969, Loomis and Spath 1969, Hoffmann 1990, Estébanes-González and Cervantes 2005); Panama (Brennan and Yunker 1966); Surinam (Brennan and van Bronswijk 1975; Brennan and Lukoschus 1971); Trinidad (Brennan and Jones 1960) and Venezuela (Brennan and Reed 1974).

**23. *Eutrombicula batatas* (Linnaeus, 1758): 617**; Surinam, ex unknown host; NHMUK.

**Records from Brazil.** This species was found in Manaus and Carvoeiro (Amazonas) parasitizing *G. domesticus*, and in Belém (Pará), parasitizing *Meleagris gallopavo* (Galliformes) (Ewing 1925). There are also records of *E. batatas* parasitizing *Dasyprocta agouti* (Rodentia) and unidentified Cervidae in Pará state (Bequaert 1926). Confalonieri and De Carvalho (1973) found *E. batatas* parasitizing *G. domesticus* in Mangaratiba (Rio de Janeiro). This species was also found parasitizing equines in Cáceres (Mato Grosso) (Confalonieri and Benez 1976) and on goats in Maranhão state (Faccini et al. 2017).

**Other records.** Argentina (Brennan and Jones 1964b); Bolivia (Brennan 1970a); Costa Rica (Arnold 1970); Curaçao (Brennan 1967); Guatemala (Brennan and Dalmat 1960); Jamaica (Brennan 1953); Mexico (Linnaeus 1758, Jenkins 1949, Loomis and Stephens 1962, Loomis 1969, Hoffmann 1990, Estébanes-González and Cervantes 2005); Panama (Brennan and Yunker 1966); Surinam (Linnaeus 1758, Brennan and Lukoschus 1971, Brennan and van Bronswijk 1975); Trinidad (Brennan and Jones 1960, Bennett and Loomis 1980) and Venezuela (Brennan and Reed 1974).

**24. *Eutrombicula bruyanti* (Oudemans, 1910): 85**; southern Brazil; ex *Didelphis* sp. “opossum”; RMNH.

**Records from Brazil.** Only the type data, this species was described from an unknown location in southern Brazil.

**25. *Eutrombicula goeldii* (Oudemans, 1910): 84**; Brazil; ex *Dasyprocta aguti*; RMNH.

**Records from Brazil.** Only the type data, no locality information was provided by the author.

**Other records.** Bolivia (Brennan 1970a); Colombia (Boshell and Kerr 1942, Brennan 1968a); Costa Rica (Arnold 1970); Dominica (Brennan 1967); Panama (Brennan and Yunker 1966); Surinam (Brennan 1970c, Brennan and Lukoschus 1971, Brennan and van Bronswijk 1975); Trinidad (Brennan and Jones 1960); Venezuela (Brennan and Reed 1974).

**26. *Eutrombicula tinami* (Oudemans, 1910): 84**; Brazil; ex *Crypturus noctivagus*; RMNH.

**Records from Brazil.** Only the type data, no locality information was provided by the author.

**New record.** IBSP 11368 (8 larvae), Condomínio Vila Verde, Itapevi, São Paulo, state of São Paulo, 19-VI-2013, *Didelphis aurita* (#VV 107).

**Other records.** Panama (Brennan and Lukoschus 1971); Surinam (Brennan and Lukoschus 1971); and Venezuela (Brennan and Reed 1974).

**Genus *Fonsecia* Radford, 1942: 56**

**27. *Fonsecia ewingi* (Fonseca, 1932): 153**; Correntes, Mato Grosso state, Brazil, ex *Xenodon merremii*; IBSP (syntypes, n° 27).

**Records from Brazil.** In addition to the type data, this species was also found at the following localities: Quiteriozinho (Mato Grosso), Birigui, Guatapará, Jacaré, Matão, Penápolis, Promissão and Silvania (São Paulo), Morrinhos (Goiás) and Rio Negro (Paraná) (Fonseca 1932b).

**Other records.** Trinidad (Brennan 1967).

**28. *Fonsecia ophidica* (Fonseca, 1932): 151**; Promissão, São Paulo state, Brazil, ex *Xenodon merremii*; IBSP (syntypes, n° 29).

**Records from Brazil.** In addition to the type data, this species was also found in Matão (São Paulo), on the same host (Fonseca 1932b).

**29. *Fonsecia travassosi* (Fonseca, 1936): 29**; Angra dos Reis, Rio de Janeiro state, Brazil; ex *Spilotes pullatus*; IBSP (holotype, n° 30).

**Records from Brazil.** Only the type data.

**Genus *Hooperella* Vercammen-Grandjean, 1967: 853**

**30. *Hooperella spinirostra* (Vercammen-Grandjean, 1967): 854**; Lagoa Santa, Minas Gerais state, Brazil, ex *Micronycteris megalotis*; USNM (holotype, n° 10462/B).

**Records from Brazil.** Only the type data.

**Genus *Kymocta* Yunker & Brennan, 1962: 572**

**31. *Kymocta brasiliensis* (Fonseca, 1936): 32**; Butantan, São Paulo, São Paulo state, Brazil, ex “wild mouse”; IBSP (holotype, n° 334).

**Records from Brazil.** Only the type data.

**New records.** IBSP 11129C (3 larvae), Morro Grande, Cotia, state of São Paulo, 26-IV-2012, *Akodon montensis* (#MGR 38).

**32. *Kymocta faitkeni* Brennan, 1968: 614**; Serra do Navio, Amapá state, Brazil, ex *Hylaeamys megacephalus* (= *Oryzomys capito*); USNM (holotype and paratypes, n° 48875) and NHMUK (paratypes).

**Records from Brazil.** In addition to the type data, this species was record in the same locality and host by Brennan and van Bronswijk (1973) and was also found in Bragança (Pará) parasitizing *Necromys lasiurus* (= *Zygodontomys lasiurus*) (Rodentia) and *Didelphis marsupialis* (Didelphimorphia) (Brennan and van Bronswijk 1973).

**Other records.** Trinidad (Brennan 1968b) and Venezuela (Brennan and Yunker 1969, Brennan and van Bronswijk 1973).

**33. *Kymocta inca* (Brennan & Jones, 1961): 177**; Quince Mil, Cuzco, Peru, *Nephelomys keaysi* (= *Oryzomys keaysi*); USNM (holotype and paratypes, n° 33554).

**Records from Brazil.** In addition to the type data, this species was record in the same locality and host by Brennan and van Bronswijk (1973) and on the rodent *P. guyannensis* in Bragança (Pará) (Brennan and van Bronswijk 1973).

**Other records.** Peru (Brennan and Jones 1961a) and Venezuela (Brennan and Yunker 1969).

**34. *Kymocta lutui* Goff, Whitaker & Dietz, 1983: 185**; National Park of Serra da Canastra, Minas Gerais state, Brazil, *Necromys lasiurus* (= *Zygodontomys lasiurus*); BPBM (holotype and paratypes, n°12712).

**Records from Brazil.** Only the type data.

**Genus *Microtrombicula* Ewing, 1950: 297**

**35. *Microtrombicula brachytrichia* Brennan, 1971: 214**; Belém, Para state, Brazil, *Proechimys guyannensis*; USNM (holotype and paratypes n° 50350).

**Records from Brazil.** Only the type data.

**36. *Microtrombicula brennani* Goff, Whitaker & Dietz, 1986: 171**; Poço das Antas Biological Reserve, Rio de Janeiro state, Brazil, ex *Lentopithecus rosalia*; USNM (holotype and paratypes) and BPBM (paratypes).

**Records from Brazil.** Only the type data.

**37. *Microtrombicula rhipidomysi* Goff, Whitaker & Dietz, 1983: 183**; National Park of Serra da Canastra, state of Minas Gerais, Brazil, ex *Rhipidomys mastacalis*; BPBM (holotype, n° 12710 and paratypes)

**Records from Brazil.** Only the type data.

**Genus *Neoschoengastia* Ewing, 1929: 187**

**38. *Neoschoengastia esorhina* Brennan, 1971: 666**; Ananindeua, Pará state, Brazil, ex *Automolus infuscatus*, USNM (holotype and paratypes, n° 56193).

**Records from Brazil.** Only the type data.

**Genus *Parascoshoengastia* Vercammen-Grandjean, 1960: 469**

**39. *Parascoschoengastia aemulata* (Brennan & Jones, 1964): 307**; Rancho Grande, Aragua, Venezuela, ex *Anoura caudata*; USNM (holotype and paratypes, n° 40658), FMNH (paratypes), OSAL (paratypes), BPNM (paratypes).

**Records from Brazil.** Present study.

**New record.** IBSP 12558 (1 larva), Morro Grande, Cotia, São Paulo, 20-X-2015, *Akodon* sp. (#MGR 246).

**Other records.** Venezuela (Brennan and Jones 1964a).

**Genus *Parasecia* Loomis, 1966: 191**

**40. *Parasecia aitkeni* (Brennan & Jones, 1960): 510**; Cumaca, Trinidad, ex *Nectomys squamipes*; USNM (holotype and paratypes, n° 33628), FHMUK (paratypes), FMNH (paratypes) and SAM (paratypes).

**Records from Brazil.** In addition to the type data, this species was found in the same locality, but parasitizing the marsupial *Monodelphis domestica* (Whitaker and Dietz 1987).

**Other records.** Bolivia (Brennan 1970a); Surinam (Brennan and Lukoschus 1971); Trinidad (Brennan and Jones 1960, Brennan 1969c) and Venezuela (Brennan and Reed 1975).

**41. *Parasecia fundata* Brennan, 1969: 664**; Belém, Pará state, and Serra do Navio, Amapá state, Brazil, ex *Caluromys philander*, *Didelphis marsupialis* and *Glyphorynchus spirurus*; USNM (holotype and paratypes, n° 49323) and NHMUK (paratypes).

**Records from Brazil.** In addition to the type data, this species was also found parasitizing *P. guyannensis* in Serra do Navio (Amapá) (Brennan 1969c).

**Other records.** Costa Rica (Stekolnikov et al. 2007).

**42. *Parasecia lasiurus* Goff & Gettinger, 1991: 401**; Brasília, Federal District, Brazil, ex *Necromys lasiurus* and *Oxymycterus* sp.; MZSP (holotype), BPBM (paratypes), OMNH (paratypes).

**Records from Brazil.** Only the type data.

**43. *Parasecia orphana* Brennan, 1971: 212**; Belém, Pará state, Brazil, ex *Proechimys guyannensis*; USNM (holotype and paratypes, n° 50351), NHMUK (paratypes), FMNH (paratypes).

**Records from Brazil.** Only the type data.

**44. *Parasecia thalurania* Brennan, 1969: 663**; Belém, Pará state, Brazil; ex *Thalurania furcata*; USNM (holotype, n°49374).

**Records from Brazil.** Only the type data.

**45. *Parasecia valida* Brennan, 1969: 663**; Bragança, Pará state, and Serra do Navio, Amapá state, Brazil, ex *Hylaeamys megacephalus*, *Necromys lasiurus* (= *Zygodontomys lasiurus fuscinus*) and *Monodelphis brevicaudata*; USNM (holotype and paratypes, n° 49092) and NHMUK (paratypes).

**Records from Brazil.** Only the type data.

**Other records.** Surinam (Brennan and Lukoschus 1971).

**Genus *Paratrombicula* Goff & Whitaker, 1984: 329**

**46. *Paratrombicula plaumanni* (Brennan & Jones, 1964): 309**; Nova Teutônia, Santa Catarina state, Brazil, ex *Ctenomys minutus*; USNM (holotype and paratypes, n° 34911), NHMUK (paratypes), FMNH (paratypes), OSAL (paratypes), BPNM (paratypes).

**Records from Brazil.** Only the type data.

**Other records.** Chile (Stekolnikov and González-Acuña 2012).

**Genus *Perissopalla* Brennan & White, 1960: 303**

**47. *Perissopalla barticonycteris* Brennan, 1969: 429**; Belém, Pará state, Brazil, ex *Glyphonycteris daviesi*; USNM (holotype and paratypes, n° 49939).

**Records from Brazil.** Only the type data.

**Other records.** Bolivia (Brennan 1970a) and Surinam (Brennan and Lukoschus 1971, Brennan and van Bronswijk 1975).

**48. *Perissopalla ipeani* Brennan, 1969: 429**; Belém, Pará state, Brazil, ex *Carollia perspicillata*; USNM (holotype and paratypes, n° 49366).

**Records from Brazil.** In addition to the type data, this species was also reported from *Tonatia bidens* (Chiroptera) in Parque Estadual Pedra Branca (Rio de Janeiro) (Almeida et al. 2011).

**Other records.** Surinam (Brennan and van Bronswijk 1975, Brennan and Lukoschus 1971).

**49. *Perissopalla tanycera* Brennan, 1969: 430**; Icabarú, Bolivar, Venezuela, ex *Peropteryx* sp.; RML (holotype and paratypes, n° 49833).

**Records from Brazil.** This species was found in Príncipe de Beira (Rondônia) parasitizing the bat *Saccopteryx bilineata* (Brennan 1970a).

**Other records.** Bolivia (Brennan 1970a); Surinam (Brennan and van Bronswijk 1975) and Venezuela (Brennan 1969b, Brennan and Reed 1975).

**Genus *Polylopadium* Brennan & Jones, 1961: 112**

**50. *Polylopadium aspasium* Brennan, 1969: 868**; Serra do Navio, Amapá state and Bragança, Pará state, Brazil; *ex H. megacephalus* (= *Oryzomys capito*); RML (holotype and paratypes, n° 49341).

**Records from Brazil.** In addition to the type data, this species was found in the same host, but in Belém (Pará) (Brennan 1969a).and also was found on the rodent *Proechimys* sp.in Príncipe da Beira (Rondônia) (Brennan 1970a).

**Other records.** Venezuela (Brennan and Reed 1975).

**Genus *Quadraseta* Brennan, 1970: 1695**

**51. *Quadraseta brasiliensis* Goff & Gettinger, 1989: 557**; Brasília, Federal District, Brazil, ex *Hylaeamys megacephalus*, *Gracilinannus agilis*, *Monodelphis Americana*; MZSP (holotype), BPBM (paratypes) and OMNH (paratypes).

**Records from Brazil.** Only the type data.

**52. *Quadraseta flochi* (Brennan & Jones, 1960): 503**; Maingot state, Trinidad, ex *Rattus rattus*; USNM (holotype and paratypes, n° 33885), FMNH (paratypes), NHMUK (paratypes) and SAM (paratypes).

**Records from Brazil.** Present study.

**New record.** IBSP 11096F (2 larvae), Morro Grande, Cotia, São Paulo state, 20-VI-2012, *Euryoryzomys russatus* (#MGR 32).

**Other records.** Colombia (Brennan 1968a), Surinam (Brennan and Lukoschus 1971), Trinidad (Brennan and Jones 1960) and Venezuela (Brennan and Reed 1975).

**53. *Quadraseta mirandae* Goff & Brennan, 1977: 504**; Quebrada Chacaito, Miranda, Venezuela, ex *Nephelomys albigularis* (= *Oryzomys albigularis*); USNM (holotype and paratypes, n° 52518) and BPBM (paratypes).

**Records from Brazil.** Present study.

**New records.** IBSP 10606C (1 larva), Morro Grande, Cotia, São Paulo state, 13-IX-2011, *Akodon montensis* (#MGR 6); 1 larva (IBSP 11129B), same locality and host (#MGR 38), 26-VI-2012.

**Other records.** Venezuela (Goff and Brennan 1977).

**54. *Quadraseta pazca* (Brennan & Jones, 1964): 700**; Azul, Buenos Aires, Argentina, ex *Rattus* sp. and *Mus* sp.; USNM (holotype and paratypes, n° 38090), NHMUK (paratypes), FMNH (paratypes) and OSAL (paratypes).

**Records from Brazil.** Whitaker and Dietz (1987) reported this species from the Serra da Canastra National Park (Minas Gerais) on the rodent *Calomys tener*.

**New records.** IBSP 10606B (1 larva), Morro Grande, Cotia, São Paulo state, 13-IX-2011, *Akodon montensis* (#MGR 6); 2 larvae (IBSP 11096E), same locality, 20-VI-2012, *Euryoryzomys russatus* (#MGR 32).

**Other records.** Argentina (Brennan and Jones 1964b).

**55. *Quadraseta trapezoides* (Brennan & Jones, 1964): 699**; Azul, Buenos Aires, Argentina, ex “fox”; USNM (holotype and paratypes, n° 38095), NHMUK (paratypes), FMNH (paratypes) and OSAL (paratypes).

**Records from Brazil.** Present study.

**New record.** IBSP 11110C (12 larvae), Morro Grande, Cotia, São Paulo state, 16-VII-2012, *Nectomys squamipes* (#MGR 40).

**Other records.** Argentina (Brennan and Jones 1964b).

**Genus *Rhinibius* Brennan & Yunker, 1969: 304**

**56. *Rhinibus tamandua* Brennan & Yunker, 1969: 304**; Belém, Pará state, ex *Tamandua tetradactyla*; USNM (holotype and paratypes, n° 49365), FMNH (paratypes) and NHMUK (paratypes).

**Records from Brazil.** Only the type data.

**Other records.** Venezuela (Brennan and Reed 1975, Brennan and Yunker 1969).

**Genus *Serratacarus* Goff & Whitaker, 1984: 162**

**57. *Serratacarus dietzi* Goff & Whitaker, 1984: 163**; Serra da Canastra National Park, Minas Gerais state, Brazil; ex *Necromys lasiurus*; USNM (holotype and paratypes) and BPBM (paratypes).

**Records from Brazil.** Only the type data.

**58. *Serratacarus lasiurus* Goff & Whitaker, 1984: 166**; Serra da Canastra National Park, Minas Gerais state, Brazil; ex *Necromys lasiurus*; USNM (holotype and paratypes) and BPBM (paratypes).

**Records from Brazil.** Only the type data.

**Genus *Speleocola* Lipovsky, 1952: 132**

**59. *Speleocola tamarina* Goff, Whitaker & Dietz, 1987: 198**; Poço das Antas Biological Reserve, Rio de Janeiro state, Brazil, ex *Lentopithecus rosalia*; USNM (holotype and paratypes).

**Records from Brazil.** The type data and the present study.

**New records.** IBSP 11364B (1 larva), Morro Grande, Cotia, São Paulo, 21-VII-2013, *Sooretamys angouya* (#MGR 148).

**Genus *Trombewingia* Fonseca, 1955: 3**

**60. *Trombewingia bakeri* (Fonseca, 1955): 3**; Reserva Florestal do Horto Florestal de São Paulo, São Paulo state, Brazil, ex *Guerlinguetus ingrami*; IBSP (Lectotype and Paralectotypes, n° 344).

**Records from Brazil.** In addition to the type data, Whitaker and Dietz (1987) reported it from the Serra da Canastra National Park (Minas Gerais) parasitizing the marsupial *M. domestica*. Other recent reports from São Paulo state include the municipality of Campos do Jordão and municipality of Cotia parasitizing *A. montensis*, and the rodents *Delomys dorsalis* and *Sooretamys angouya* (Jacinavicius et al. 2015).

**61. *Trombewingia brasiliensis* Goff & Gettinger, 1991: 401**; Brasília, Federal District, Brazil, ex *Necromys lasiurus* (= *Bolomys lasiurus*); MZSP (holotype), BPBM (paratypes) and OMNH (paratypes).

**Records from Brazil.** Only the type data.

**Genus *Trombicula* Berlese, 1905: 155**

**62. *Trombicula truncata* Brennan, 1970: 272**; Príncipe de Beira, Rondônia state, Brazil, ex *Saccopteryx bilineata*; USNM (holotype and paratypes, n° 50188), FMNH (paratypes) and NHMUK (paratypes).

**Records from Brazil.** Only the type data.

**Genus *Vercammenia* Audy & Nadchatram, 1957: 95**

**63. *Vercammenia yorkei* (Sambon, 1928): 119**; Urucum, Mato Grosso do Sul state, Brazil, ex *Scinax funereus* (= *Hyla rubra*); NHMUK (holotype, n° 147-9).

**Records from Brazil.** Only the type data.

## Appendix 1

Taxonomic list of chiggers and their respective hosts.

**Leeuwenhoekiidae**

***Apolonia tigipioensis* Torres & Braga, 1938**

Aves

Galliformes

Phasianidae

*Gallus gallus domesticus*

Passeriformes

Passeridae

*Passer domesticus*

Struthioniformes

Struthionidae

*Struthio camelus*

Tinamiformes

Tinamidae

*Nothura maculosa cearensis*

Mammalia

Primates

Hominidae

*Homo sapiens*

***Hannemania hepatica* Fonseca, 1936**

Amphibia

Anura

Leptodactylidae

*Leptodactylus latrans*

***Hannemania hylodeus* (Oudemans, 1910)**

Amphibia

Anura

Hylodidae

*Hylodes* sp.

***Hannemania newsteadi* Sambon, 1928**

Amphibia

Anura

Hylidae

*Scinax funereus*

***Hannemania stephensi* Sambon, 1928**

Amphibia

Anura

Craugastoridae

*Pristimantis conspicillatus*

***Leeuwenhoekia verduni* (Oudemans, 1910)**

Mammalia

Didelphimorphia

Didelphidae

*Didelphis* sp.

***Whartonia nudosetosa* (Wharton, 1938)**

Mammalia

Chiroptera

Phyllostomidae

*Carollia perspicillata*

***Whartonia pachywhartoni* Vercammen-Grandjean, 1966**

Mammalia

Chiroptera

Phyllostomidae

*Carollia perspicillata*

*Micronycteris megalotis*

**Trombiculidae**

***Aitkenius vellosus* Brennan, 1970**

Mammalia

Rodentia

Cricetidae

*Hylaeamys megacephalus*

*Nectomys squamipes*

Echimyidae

*Proechimys guyannensis*

***Arisocerus amapensis* Brennan, 1970**

Mammalia

Rodentia

Cricetidae

*Euryoryzomys macconnelli*

*Hylaeamys megacephalus*

Echimyidae

*Proechimys guyannensis*

***Arisocerus hertigi* (Brennan & Jones, 1964)**

Mammalia

Didelphimorphia

Didelphidae

*Didelphis albiventris*

Rodentia

Caviidae

*Cavia intermedia*

***Blankaartia shatrovi*Bassini-Silva & Barros-Battesti, 2016**

Aves

“bird”

Passeriformes

Thraupidae

*Trichothraupis melanops*

***Blankaartia sinnamaryi* Floch & Fauran, 1956**

Aves

Passeriformes

Conopophagidae

*Conopophaga lineata*

Pipridae

*Manacus manacus*

Thamnophilidae

*Thamnophilus caerulescens*

Thraupidae

*Tachyphonus coronatus*

Turdida

*Turdus albicollis*

*Turdus rufiventris*

Piciformes

Picidae

*Picumnus cirratus*

Mammalia

Primates

Hominidae

*Homo sapiens*

***Buclypeus catatonus* Brennan, 1972**

Mammalia

Rodentia

Echimyidae

*Proechimys guyannensis*

***Buclypeus ignotus* (Brennan, 1971)**

Mammalia

Rodentia

Echimyidae

*Proechimys guyannensis*

***Caamembecaia gratiosus* Gazêta, Amorim, Bossi, Linhares & Serra-Freire, 2006**

Mammalia

Rodentia

Echimyidae

*Trinomys gratiosus*

**Chiroptella (Oudemansidium) australis (Brennan, 1970)**

Mammalia

Chiroptera

Molossidae

*Nyctinomops laticaudatus*

***Colicus brasiliensis* Goff, Whitaker & Dietz, 1983**

Mammalia

Rodentia

Cricetidae

*Cerradomys subflavus*

*Oligoryzomys fornesi*

***Colicus icomi* Brennan, 1970**

Mammalia

Rodentia

Echimyidae

*Proechimys guyannensis*

***Colicus spinosus* Goff & Gettinger, 1989**

Mammalia

Didelphimorphia

Didelphidae

*Gracilinannus agilis*

*Monodelphis* sp.

***Euschoengastia trouessarti* (Oudemans, 1910)**

Mammalia

Didelphimorphia

Didelphidae

*Didelphis* sp.

***Eutrombicula alfreddugesi* Oudemans, 1910**

Mammalia

Artiodactyla

Bovidae

“goats”

Primates

Hominidae

*Homo sapiens*

Reptilia

Squamata

Colubridae

*Xenodon merremii*

Tropiduridae

*Tropidurus cocorobensis*

*Tropidurus erythrocephalus*

*Tropidurus hispidus*

*Tropidurus itambere*

*Tropidurus oreadicus*

*Tropidurus semitaeniatus*

*Tropidurus torquatus*

***Eutrombicula batatas* (Linnaeus, 1758)**

Aves

Galliformes

Phasianidae

*Gallus domesticus*

*Meleagris gallopavo*

Mammalia

Artiodactyla

Cervidae

Unidentified

Bovidae

“goats”

Perissodactyla

Equidae

“Equines”

Rodentia

Dasyproctidae

*Dasyprocta agouti*

***Eutrombicula bruyanti* (Oudemans, 1910)**

Mammalia

Didelphimorphia

Didelphidae

*Didelphis* sp.

***Eutrombicula goeldii* (Oudemans, 1910)**

Mammalia

Rodentia

Dasyproctidae

*Dasyprocta agouti*

***Eutrombicula tinami* (Oudemans, 1910)**

Aves

Tinamiformes

Tinamidae

*Crypturellus noctivagus*

Mammalia

Didelphimorphia

Didelphidae

*Didelphis aurita*

***Fonsecia ewingi* (Fonseca, 1932)**

Reptilia

Squamata

Colubridae

*Xenodon merremii*

***Fonsecia ophidica* (Fonseca, 1932)**

Reptilia

Squamata

Colubridae

*Xenodon merremii*

***Fonsecia travassosi* (Fonseca, 1936)**

Reptilia

Squamata

Colubridae

*Spilotes pullatus*

***Hooperella spinirostra* (Vercammen-Grandjean, 1967)**

Mammalia

Chiroptera

Phyllostomidae

*Micronycteris megalotis*

***Kymocta brasiliensis* (Fonseca, 1936)**

Mammalia

Rodentia

“wild mouse”

Cricetidae

*Akodon montensis*

***Kymocta faitkeni* Brennan, 1968**

Mammalia

Didelphimorphia

Didelphidae

*Didelphis marsupialis*

Rodentia

Cricetidae

*Hylaeamys megacephalus*

*Necromys lasiurus*

***Kymocta inca* (Brennan & Jones, 1961)**

Mammalia

Rodentia

Cricetidae

*Hylaeamys megacephalus*

Echimyidae

*Proechimys guyannensis*

***Kymocta lutui* Goff, Whitaker & Dietz, 1983**

Mammalia

Rodentia

Cricetidae

*Necromys lasiurus*

***Microtrombicula brachytrichia* Brennan, 1971**

Mammalia

Rodentia

Echimyidae

*Proechimys guyannensis*

***Microtrombicula brennani* Goff, Whitaker & Dietz, 1986**

Mammalia

Primates

Callitrichidae

*Lentopithecus rosalia*

***Microtrombicula rhipidomysi* Goff, Whitaker & Dietz, 1983**

Mammalia

Rodentia

Cricetidae

*Rhipidomys mastacalis*

***Neoschoengastia esorhina* Brennan, 1971**

Aves

Passeriformes

Furnariidae

*Automolus infuscatus*

***Parascoschoengastia aemulata* (Brennan & Jones, 1964)**

Mammalia

Rodentia

Cricetidae

*Akodon* sp.

***Parasecia aitkeni* (Brennan & Jones, 1960)**

Mammalia

Didelphimorphia

Didelphidae

*Monodelphis domestica*

Rodentia

Cricetidae

*Calomys tener*

*Nectomys squamipes*

***Parasecia fundata* Brennan, 1969**

Aves

Passeriformes

Dendrocolaptidae

*Glyphorynchus spirurus*

Mammalia

Didelphimorphia

Didelphidae

*Caluromys philander*

*Didelphis marsupialis*

Rodentia

Echimyidae

*Proechimys guyannensis*

***Parasecia lasiurus* Goff & Gettinger, 1991**

Mammalia

Rodentia

Cricetidae

*Necromys lasiurus*

*Oxymycterus* sp.

***Parasecia orphana* Brennan, 1971**

Mammalia

Rodentia

Echimyidae

*Proechimys guyannensis*

***Parasecia thalurania* Brennan, 1969**

Aves

Apodiformes

Trochilidae

*Thalurania furcata*

***Parasecia valida* Brennan, 1969**

Mammalia

Didelphimorphia

Didelphidae

*Monodelphis brevicaudata*

Rodentia

Cricetidae

*Hylaeamys megacephalus*

*Necromys lasiurus*

***Paratrombicula plaumanni* (Brennan & Jones, 1964)**

Mammalia

Rodentia

Ctenomyidae

*Ctenomys minutus*

***Perissopalla barticonycteris* Brennan, 1969**

Mammalia

Chiroptera

Phyllostomidae

*Glyphonycteris daviesi*

***Perissopalla ipeani* Brennan, 1969**

Mammalia

Chiroptera

Phyllostomidae

*Carollia perspicillata*

*Tonatia bidens*

***Perissopalla tanycera* Brennan, 1969**

Mammalia

Chiroptera

Emballonuridae

*Saccopteryx bilineata*

***Polylopadium aspasium* Brennan, 1969**

Mammalia

Rodentia

Cricetidae

*Hylaeamys megacephalus*

Echimyidae

*Proechimys* sp.

***Quadraseta brasiliensis* Goff & Gettinger, 1989**

Mammalia

Didelphimorphia

Didelphidae

*Gracilinannus agilis*

*Monodelphis americana*

Rodentia

Cricetidae

*Hylaeamys megacephalus*

***Quadraseta flochi* (Brennan & Jones, 1960)**

Mammalia

Rodentia

Cricetidae

*Euryoryzomys russatus*

***Quadraseta mirandae* Goff & Brennan, 1977**

Mammalia

Rodentia

Cricetidae

*Akodon montensis*

***Quadraseta pazca* (Brennan & Jones, 1964)**

Mammalia

Rodentia

Cricetidae

*Euryoryzomys russatus*

*Calomys tener*

***Quadraseta trapezoides* (Brennan & Jones, 1964)**

Mammalia

Rodentia

Cricetidae

*Nectomys squamipes*

***Rhinibus tamandua* Brennan & Yunker, 1969**

Mammalia

Pilosa

Myrmecophagidae

*Tamandua tetradactyla*

***Serratacarus dietzi* Goff & Whitaker, 1984**

Mammalia

Rodentia

Cricetidae

*Nectomys lasiurus*

***Serratacarus lasiurus* Goff & Whitaker, 1984**

Mammalia

Rodentia

Cricetidae

*Nectomys lasiurus*

***Speleocola tamarina* Goff, Whitaker & Dietz, 1987**

Mammalia

Primates

Callitrichidae

*Lentopithecus rosalia*

Rodentia

Cricetidae

*Sooretamys angouya*

***Trombewingia bakeri* (Fonseca, 1955)**

Mammalia

Rodentia

Cricetidae

*Akodon montensis*

*Delomys dorsalis*

*Sooretamys angouya*

Sciuridae

*Guerlinguetus ingrami*

Didelphimorphia

Didelphidae

*Monodelphis domestica*

***Trombewingia brasiliensis* Goff & Gettinger, 1991**

Mammalia

Rodentia

Cricetidae

*Necromys lasiurus* (= *Bolomys lasiurus*)

***Trombicula truncata* Brennan, 1970**

Mammalia

Chiroptera

Emballonuridae

*Saccopteryx bilineata*

***Vercammenia yorkei* (Sambon, 1928)**

Amphibia

Anura

Hylidae

*Scinax funereus*

## Appendix 2

List of hosts, their associations with chiggers and the respective reference.

**Amphibia**

Anura

Craugastoridae

*Pristimantis conspicillatus*

*Hannemania stephensi* Sambon 1928

Hylidae

*Scinax funereus*

*Hannemania newsteadi* Sambon 1928

*Vercammenia yorkei* Sambon 1928

Hylodidae

*Hylodes* sp.

*Hannemania hylodeus* Oudemans 1910

Leptodactylidae

*Leptodactylus latrans*

*Hannemania hepatica* Fonseca 1936

**Reptilia**

Squamata

Colubridae

*Spilotes pullatus*

*Fonsecia travassosi* Fonseca 1936

*Xenodon merremii*

*Eutrombicula alfreddugesi* Fonseca 1932a

*Fonsecia ewingi* Fonseca 1932b

*Fonsecia ophidica* Fonseca 1932b

Tropiduridae

*Tropidurus cocorobensis*

*Eutrombicula alfreddugesi* Menezes et al. 2011

*Tropidurus erythrocephalus*

*Eutrombicula alfreddugesi* Menezes et al. 2011

*Tropidurus hispidus*

*Eutrombicula alfreddugesi* Delfino et al. 2011, Menezes et al. 2011

*Tropidurus itambere*

*Eutrombicula alfreddugesi* Carvalho et al. 2006

*Tropidurus oreadicus*

*Eutrombicula alfreddugesi* Carvalho et al. 2006

*Tropidurus semitaeniatus*

*Eutrombicula alfreddugesi* Menezes et al. 2011

*Tropidurus torquatus*

*Eutrombicula alfreddugesi* Carvalho et al. 2006

**Aves**

“bird”

*Blankaartia shatrovi* Bassini-Silva et al. 2016

Apodiformes

Trochilidae

*Thalurania furcata*

*Parasecia thalurania* Brennan 1969c

Galliformes

Phasianidae

*Gallus domesticus*

*Apolonia tigipioensis* Torres and Braga 1938

*Eutrombicula batatas* Ewing 1925, Confalonieri and De Carvalho 1973

*Meleagris gallopavo*

*Eutrombicula batatas* Ewing 1925

Passeriformes

Conopophagidae

*Conopophaga lineata*

*Blankaartia sinnamaryi* Bassini-Silva et al. 2016

Dendrocolaptidae

*Glyphorynchus spirurus*

*Parasecia fundata* Brennan 1969c

Furnariidae

*Automolus infuscatus*

*Neoschoengastia esorhina* Brennan 1971a

Pipridae

*Manacus manacus*

*Blankaartia sinnamaryi* Bassini-Silva et al. 2016

Passeridae

*Passer domesticus*

*Apolonia tigipioensis* Ornelas-Almeida et al. 2007

Thamnophilidae

*Thamnophilus caerulescens*

*Blankaartia sinnamaryi* Bassini-Silva et al. 2016

Thraupidae

*Tachyphonus coronatus*

*Blankaartia sinnamaryi* Bassini-Silva et al. 2016

*Trichothraupis melanops*

*Blankaartia shatrovi* Bassini-Silva et al. 2016

Turdidae

*Turdus albicollis*

*Blankaartia sinnamaryi* Bassini-Silva et al. 2016

*Turdus rufiventris*

*Blankaartia sinnamaryi* Bassini-Silva et al. 2016

Piciformes

Picidae

*Picumnus cirratus*

*Blankaartia sinnamaryi* Bassini-Silva et al. 2016

Struthioniformes

Struthionidae

*Struthio camelus*

*Apolonia tigipioensis* Ornelas-Almeida et al. 2007

Tinamiformes

Tinamidae

*Crypturellus noctivagus*

*Eutrombicula tinami* Oudemans 1910

*Nothura maculosa cearensis*

*Apolonia tigipioensis* Carneiro 1949, 1952

**Mammalia**

Artiodactyla

Bovidae

Goats

*Eutrombicula batatas* Faccini et al. 2017

*Eutrombicula alfreddugesi* Faccini et al. 2017

Cervidae

Unidentified

*Eutrombicula batatas* Bequaert 1926

Chiroptera

Emballonuridae

*Saccopteryx bilineata*

*Perissopalla tanycera* Brennan 1970a

*Trombicula truncata* Brennan 1970a

Phyllostomidae

*Carollia perspicillata*

*Perissopalla ipeani* Brennan 1969b

*Whartonia nudosetosa* Silveira et al. 2015

*Whartonia pachywhartoni* Silveira et al. 2015

*Glyphonycteris daviesi*

*Perissopalla barticonycteris* Brennan 1969b

*Micronycteris megalotis*

*Hooperella spinirostra* Vercammen-Grandjean 1967

*Whartonia pachywhartoni* Vercammen-Grandjean 1966

*Tonatia bidens*

*Perissopalla ipeani* Almeida et al. 2011

Molossidae

*Nyctinomops laticaudatus*

Chiroptella (Oudemansidium) australis Brennan 1970a

Didelphimorphia

Didelphidae

*Caluromys philander*

*Parasecia fundata* Brennan 1969c

*Didelphis albiventris*

*Arisocerus hertigi* Goff and Gettinger 1989

*Didelphis aurita*

*Eutrombicula tinami* this study

*Didelphis marsupialis*

*Kymocta faitkeni* Brennan and Bronswijk 1973

*Parasecia fundata* Brennan 1969c

*Didelphis* sp.

*Euschoengastia trouessarti* Oudemans 1910

*Eutrombicula bruyanti* Oudemans 1910

*Leeuwenhoekia verduni* Oudemans 1910

*Gracilinannus agilis*

*Colicus spinosus* Goff and Gettinger 1989

*Quadraseta brasiliensis* Goff and Gettinger 1989

*Monodelphis americana*

*Quadraseta brasiliensis* Goff and Gettinger 1989

*Monodelphis brevicaudata*

*Parasecia valida* Brennan 1969c

*Monodelphis domestica*

*Parasecia aitkeni* Whitaker and Dietz 1987

*Trombewingia bakeri* Whitaker and Dietz 1987

*Monodelphis* sp.

*Colicus spinosus* this study

Perissodactyla

Equidae

“Equines” Confalonieri and Benez 1976

*Eutrombicula batatas*

Pilosa

Myrmecophagidae

*Tamandua tetradactyla*

*Rhinibus tamandua* Brennan and Yunker 1969

Primates

Callitrichidae

*Lentopithecus rosalia*

*Microtrombicula brennani* Goff et al. 1986

*Speleocola tamarina* Goff et al. 1987

Hominidae

*Homo sapiens*

*Apolonia tigipioensis* Carneiro 1949, 1952

*Eutrombicula alfreddugesi* Fonseca 1932a

Rodentia

Unknown

“wild mouse”

*Kymocta brasiliensis* Fonseca 1936

Caviidae

*Cavia intermedia*

*Arisocerus hertigi* Regolin et al. 2015

Cricetidae

*Akodon montensis*

*Kymocta brasiliensis* this study

*Quadraseta mirandae* this study

*Quadraseta pazca* this study

*Trombewingia bakeri* Jacinavicius et al. 2015

*Akodon* sp.

*Parascoschoengastia aemulata* this study

*Calomys tener*

*Parasecia aitkeni* Whitaker and Dietz 1987

*Quadraseta pazca* Whitaker and Dietz 1987

*Cerradomys subflavus*

*Colicus brasiliensis* Goff et al. 1983

*Delomys dorsalis*

*Trombewingia bakeri* Jacinavicius et al. 2015

*Euryoryzomys macconnelli*

*Arisocerus amapensis* Brennan 1970

*Euryoryzomys russatus*

*Quadraseta flochi* this study

*Quadraseta pazca* this study

*Necromys lasiurus*

*Kymocta faitkeni* Brennan and Bronswijk 1973

*Kymocta lutui* Goff et al. 1983

*Parasecia lasiurus* Goff and Gettinger 1991

*Parasecia valida* Brennan 1969c

*Serratacarus dietzi* Goff and Whitaker 1984a

*Serratacarus lasiurus* Goff and Whitaker 1984a

*Trombewingia brasiliensis* Goff and Gettinger 1991

*Nectomys squamipes*

*Aitkenius vellosus* Brennan 1970d

*Parasecia aitkeni* Brennan and Jones 1960, Whitaker and Dietz 1987

*Quadraseta trapezoides* this study

*Hylaeamys megacephalus*

*Aitkenius vellosus* Brennan 1970d

*Arisocerus amapensis* Brennan 1970c

*Kymocta faitkeni* Brennan 1968b

*Kymocta inca* Brennan 1968b, Brennan and Bronswijk 1973

*Parasecia valida* Brennan 1969c

*Polylopadium aspasium* Brennan 1969a

*Quadraseta brasiliensis* Goff and Gettinger 1989

*Oligoryzomys fornesi*

*Colicus brasiliensis* Goff et al. 1983

*Oxymycterus* sp.

*Parasecia lasiurus* Goff and Gettinger 1991

*Rhipidomys mastacalis*

*Microtrombicula rhipidomysi* Goff et al. 1983

*Sooretamys angouya*

*Speleocola tamarina* this study

*Trombewingia bakeri* Jacinavicius et al. 2015

Ctenomyidae

*Ctenomys minutus*

*Paratrombicula plaumanni* Brennan and Jones 1964a

Dasyproctidae

*Dasyprocta aguti*

*Eutrombicula batatas* Bequaert 1926

*Eutrombicula goeldii* Oudemans 1910

Echimyidae

*Proechimys guyannensis*

*Aitkenius vellosus* Brennan 1970

*Arisocerus amapensis* Brennan 1970c

*Buclypeus catatonus* Brennan 1972

*Buclypeus ignotus* Brennan 1971b

*Colicus icomi* Brennan 1970b

*Kymocta inca* Brennan and Bronswijk 1973

*Microtrombicula brachytrichia* Brennan 1971b

*Parasecia fundata* Brennan 1969c

*Parasecia orphana* Brennan 1971b

*Proechimys* sp.

*Polylopadium aspasium* Brennan 1970a

*Trinomys gratiosus*

*Caamembecaia gratiosus* Gazêta et al. 2006

Sciuridae

*Guerlinguetus ingrami*

*Trombewingia bakeri* Fonseca 1955
